# Predictive nomogram based on serum tumor markers and clinicopathological features for stratifying lymph node metastasis in breast cancer

**DOI:** 10.1186/s12885-022-10436-3

**Published:** 2022-12-19

**Authors:** Sheng-Kai Geng, Shao-Mei Fu, Hong-Wei Zhang, Yi-Peng Fu

**Affiliations:** 1 Department of Breast Surgery, The Obstetrics and Gynecology Hospital of Fudan University, 200011 Shanghai, People’s Republic of China; 2 Department of General Surgery, Zhongshan Hospital, Fudan University, 200032 Shanghai, People’s Republic of China

**Keywords:** Breast cancer, Lymph node metastasis, Prognostic nomogram, Serum tumor markers, Clinicopathological features

## Abstract

**Background:**

This study was aimed to establish the nomogram to predict patients’ axillary node status by using patients’ clinicopathological and tumor characteristic factors.

**Methods:**

A total of 705 patients with breast cancer were enrolled in this study. All patients were randomly divided into a training group and a validation group. Univariate and multivariate ordered logistic regression were used to determine the predictive ability of each variable. A nomogram was performed based on the factors selected from logistic regression results. Receiver operating characteristic curve (ROC) analysis, calibration plots and decision curve analysis (DCA) were used to evaluate the discriminative ability and accuracy of the models.

**Results:**

Logistic regression analysis demonstrated that CEA, CA125, CA153, tumor size, vascular-invasion, calcification, and tumor grade were independent prognostic factors for positive ALNs. Integrating all the predictive factors, a nomogram was successfully developed and validated. The C-indexes of the nomogram for prediction of no ALN metastasis, positive ALN, and four and more ALN metastasis were 0.826, 0.706, and 0.855 in training group and 0.836, 0.731, and 0.897 in validation group. Furthermore, calibration plots and DCA demonstrated a satisfactory performance of our nomogram.

**Conclusion:**

We successfully construct and validate the nomogram to predict patients’ axillary node status by using patients’ clinicopathological and tumor characteristic factors.

## Introduction

The standard treatment for breast cancer patients has continually improved with the development of cancer research. Patients with clinically negative node status can avoid axillary lymph node dissection (ALND) if no metastasis is found in the sentinel lymph node (SLN) [1, 2]. In patients with a positive SLN, ALND has been the standard treatment until recent times. Efforts have been made to find if ALND can be avoided in patients with positive SLN to decrease the morbidity of complication including persistent lymphedema, paresthesia in the forearm and axilla and operated arm-weakness.

Approximately 50–70% of patients with positive SLN have no additional disease in the axillary lymph node, some studies [3–5] and nomograms [5–9] including MSKCC nomogram [10] have tried to identify the subgroup of breast cancer patients to avoid the ALND. This treatment option was challenged by the International Breast Cancer Study Group (IBCSG) trial 23 − 01 [11], which demonstrate that patients with micro-metastasis in sentinel lymph nodes (SLNs) can be spared from ALND. The Z0011 trial, enrolled with clinically node-negative patients with T1-T2 tumors and 1–2 positive sentinel lymph nodes, demonstrated that ALND did not lead to disease-free survival and overall survival benefit compared with SLNB only [12, 13]. Similarly, the AMAROS trial, the latest ten-year follow-up results also showed that for a comparable patient population, there was no significant difference in the DFS or OS between the ALND and axillary radiotherapy group [14]. Some researches tried to confirm the results in patients underwent mastectomy [15, 16]. In conclusion, accumulating studies have focused on whether we can spare patients from ALND based on the results of sentinel lymph node biopsy (SLNB). However, intraoperative SLNB will prolong the time of surgery and increase the probability of complications. Furthermore, the results of intraoperative SLNB were not as reliable as we thought, previous studies [17–21] showed the false negative rate (FNR) was 14-43%. In addition, with the preoperative examination technique improving, the majority of breast cancer patients had a smaller tumor burden and lower incidence of axillary nodal metastasis at the time of diagnosis [22], reducing the benefits of ALNB. It is therefore reasonable to dig further into the question if we can find a way to predict the status of axillary lymph nodes for breast cancer patients to avoid SLNB.

Previous studies [23–25] have demonstrated that estrogen receptor (ER), progesterone receptor (PR), and human epidermal growth factor receptor 2 (HER2) are predictive factor for nodal metastasis. L. Dihge’s research [26] and several other studies [27–30] have tried to develop a nomogram for the prediction of axillary nodal status in breast cancer. However, the axillary lymph node status partially reflects the timeline of tumor development and is affected by the biological characteristic of the primary breast tumor, which can be manifested in terms of tumor marker, vascular-invasion, and calcification. Previous studies [31–34] had revealed the diagnostic value of serum tumor markers including CEA, CA125, and CA15-3 in metastatic breast cancer. S. O’Grady et Al’s research also found that calcifications may be associated with tumor invasion into the lymphatic system [35–37]. The present studies also revealed that vascular invasion was associated with higher lymph node metastasis possibility [38] and worse patients’ clinical outcome [39]. Therefore, the assessment of tumor characteristics including tumor marker, vascular-invasion, and calcification could be of great importance in revealing the extent of axillary lymph node (ALN) metastasis for patients with breast cancer, which most of studies did not mention. So clinically easily-accessible and reliable nomogram combing patients’ preoperative clinicopathological and tumor biological characteristic factors to predict the status of the axillary lymph node is urgently needed.

In our study, we aimed to establish and independently validate the nomogram to stratify patients’ axillary node status by using patients’ clinicopathological and tumor characteristic factors, in order to decide the best treatment option for patients. Firstly, we aim to predict the presence of lymph node metastasis to avoid unnecessary SLNB considering the postoperative complications. Furthermore, we sought the possibility to predict the stage of axillary lymph nodes metastasis to evaluate whether further ALND is needed in the case of positive SLNB.

## Materials and methods

### Patients

A total of 705 patients with breast cancer who underwent curative resection and axillary staging were enrolled in this study. After obtaining Ethics Committee’s approval, between January 2002 and December 2019, all patients were collected from the Zhongshan Hospital, Fudan University. Additionally, 705 patients were randomly divided into two groups, training group (509 patients) and validation group (196 patients). The inclusion and exclusion criteria for the patients are as follows: all patients were diagnosed pathologically with breast cancer; all patients enrolled in the study did not receive neoadjuvant chemotherapy; all patients underwent resection defined as a complete resection; all patients were treated with intraoperative SLNB (we use a single tracer, methylene blue, to locate sentinel lymph nodes intraoperatively); clinically N0 and some N1 stage patients enrolled in the training or validation groups were all treated with SLNB; all patients with one or more positive SLN underwent ALND; all the blood samples were obtained within 3 days before operations, all patients had complete records including baseline characteristics (including sex, age, menopause status, stage, molecular type, pre-operation routine blood test, tumor marker). In our study, ER/PR- was defined as less than 1% of breast cancer tissue immunohistochemical (IHC) nuclear staining; ER/PR low expression was defined as 1-10% IHC nuclear staining; PR high expression was defined as > 20% nuclear staining [34]; IHC Ki67 ≤ 14% was defined as low expression and Ki67 > 14% as high expression; Her-2-positive was defined as IHC 3 + or IHC 2 + with fluorescence in situ hybridization (FISH) +; Breast cancer was divided into 4 molecular subtypes : (1) the Luminal A subtype (ER+, PR high expression, HER-2-, Ki-67 low expression), (2) the Luminal B subtype (ER+, PR low expression, HER-2-, Ki-67 high expression, or ER+, PR any, HER-2+, Ki-67 any), (3) the HER-2 enriched subtype (ER-, PR-, Her-2+, Ki-67 any), and (4) the triple negative breast cancer (TNBC) subtype (ER-, PR-, Her-2-, Ki-67 any).

#### Preoperative evaluation

All patients underwent preoperative imaging examinations (including ultrasound and mammography) to evaluate breast cancer lesions’ calcification; Calcification positive was defined as the calcifications with malignant signs, such as fine sand-like, amorphous, or pleomorphic calcifications found in the same location as the cancer foci on ultrasound or mammography. Patients’ vascular invasion status was evaluated by preoperative tumor core needle biopsy (CNB), vacuum assisted breast biopsy (VABB), or excisional biopsy.

### Statistical analysis

The association of clinicopathologic characteristics between testing cohorts and validation cohorts was analyzed by using the X² test or Fisher’s exact test or t-test as appropriate. Univariate ordered logistic regression was performed to determine the association of each variable with the possibility of lymph node metastasis. All variables with *p* ≤ 0.01 (two tailed) were considered statistically significant and included in the multivariate ordered logistic regression using backward stepwise method. A nomogram for predicting ALN metastasis or four or more ALN metastases was constructed with the remaining variables by means of the “rms” package of the R software in the training group, and the results were validated using patients in the validation group. We used the receiver operating characteristic curve (ROC) analysis and calibration plots to evaluate the discriminative ability and accuracy of the models, respectively. What’s more, decision curve analysis (DCA) was used to assess the actual benefits for clinical patients. All the statistical analyses were performed by using SPSS version 24 (SPSS Inc) and R version 4.1.

## Results

### Clinicopathological profiles of the patients

All patients (*N* = 705) were randomly divided into two groups, the training group (*N* = 509) and the validation group (*N* = 196). The clinicopathological characteristics of two groups were well balanced and presented in Table 1. The median age of all patients is 55 (26–90), training group 54 (26–90 range), and validation group 56 (27–90 range). There were no significant differences in menopausal status, the count of CEA and CA153, tumor size, histology, biological subtype, tumor grade, vascular invasion, calcification, and location between two groups. Among breast cancer patients in the training group, 187 (36.7%) experienced lymph node metastasis, 74 (14.5%) of whom with 4 or more ALN metastasis. While in the validation group, there were 81 (41.3%) patients experiencing ALN metastasis, 35 (17.8%) of whom with 4 or more ALN metastasis.

**Table 1:** Patient characteristics

Characteristics	Total (*n* = 705)	Training group (*n* = 509)	Validation group (*n* = 196)	*P* value
Age, years				
Median (range)	55 (26—90)	54 (26—90)	56 (27—90)	**0.033**
Menopausal status				
Premenopausal	375 (53.2%)	280 (55.0%)	95 (48.5%)	0.119
Postmenopausal	330 (46.8%)	229 (45.0%)	101 (51.5%)	
CEA (Ug/L)				
Average	2.773 ± 3.269	2.619 ± 2.444	3.171 ± 4.775	0.071
CA 125 (Ug/L)				
Average	15.012 ± 12.939	14.701 ± 14.021	15.818 ± 9.560	**0.028**
CA 153 (Ug/L)				
Average	11.415 ± 9.838	11.061 ± 8.538	12.335 ± 12.584	0.254
Tumor size				
Average	2.620 ± 1.476	2.636 ± 1.480	2.579 ± 1.469	0.478
T category				
T1	330 (46.8%)	228 (44.8%)	102 (52.0%)	0.22
T2	337 (47.8%)	252 (49.5%)	85 (43.4%)	
T3	38 (5.4%)	29 (5.7%)	9 (4.6%)	
Histology				
Invasive ductal carcinoma	601 (85.2%)	434 (85.3%)	167 (85.2%)	0.177
Invasive lobular carcinoma	26 (3.7%)	18 (3.5%)	8 (4.1%)	
Mixed	30 (4.3%)	25 (4.9%)	3 (2.6%)	
Other	48 (6.8%)	32 (6.3%)	16 (8.2%)	
Biological subtype				
Luminal A	98 (13.9%)	70 (13.8%)	28 (14.3%)	0.604
Luminal B	449 (63.7%)	325 (63.9%)	124 (63.3%)	
Her-2 amplified	71 (10.1%)	55 (10.8%)	16 (8.2%)	
TNBC	87 (12.3%)	59 (11.6%)	28 (14.3%)	
Tumor grade				
Well differentiated, grade 1	78 (11.1%)	51 (10.0%)	27 (13.8%)	0.341
Moderately differentiated, grade 2	297 (42.7%)	215 (42.2%)	82 (41.8%)	
Poorly differentiated, grade 3	330 (46.8%)	243 (47.7%)	87 (44.4%)	
Vascular invasion				
No	594 (84.3%)	428 (84.1%)	166 (84.7%)	0.843
Yes	111 (15.7%)	81 (15.9%)	30 (15.3%)	
Calcification				
No	331 (47.0%)	244 (47.9%)	87 (44.4%)	0.716
Yes	374 (53.0%)	265 (52.1%)	109 (55.6%)	
Lymph node metastasis				
No	437 (62.0%)	322 (63.3%)	115 (58.7%)	0.261
Yes	268 (38.0%)	187 (36.7%)	81 (41.3%)	
Pathologic N category				
N0	437 (62.0%)	322 (63.3%)	115 (58.7%)	0.328
N1	159 (22.6%)	113 (22.2%)	46 (23.5%)	
N2	65 (9.2%)	41 (8.1%)	24 (12.2%)	
N3	44 (6.2%)	33 (6.5%)	11 (5.6%)	
ER status				
No	160 (22.7%)	116 (22.8%)	44 (22.4%)	0.923
Yes	545 (77.3%)	393 (77.2%)	152 (77.6%)	
PR status				
No	261(37.0%)	192 (37.7%)	69 (35.2%)	0.535
Yes	444(63.0%)	317 (62.3%)	127 (64.8%)	
HER-2 status				
Negative	510 (72.3%)	361 (70.9%)	149 (76.0%)	0.175
Overexpressed	195 (27.7%)	148 (29.1%)	47 (24.0%)	
Location				
UOQ	422 (59.9%)	294(57.8%)	128(65.3%)	0.28
LOQ	71 (10.1%)	55(10.8%)	16(8.2%)	
LIQ	76 (10.8%)	60(11.8%)	16(8.2%)	
UIQ	114 (16.2%)	82(16.1%)	32(16.3%)	
Central	22 (3.1%)	18(3.5%)	4(2.0%)	

*Her-2* Human epidermal growth factor receptor-2, *TNBC* Triple negative breast cancer, *LIQ* Lower-inner quadrant, *LOQ* Lower-outer quadrant, *UIQ* Upper-inner quadrant, *UOQ* Upper-outer quadrant

### Independent predictive factors for positive ALNs

In this study, Univariate logistic regression analysis of the clinicopathological characteristics revealed that for the training cohort, age, CEA, CA125, CA153, tumor size, vascular-invasion, calcification, and tumor grade were significantly associated with ALNs metastasis, showed in Table 2. Histology subtype, biological subtype and location were not identified as a predictor of lymph node metastasis in univariable logistic regression analysis and were not included in the multivariable logistic regression analyses. Multivariate logistic regression analysis demonstrated that CEA (Odds ratio 1.085, 95% CI 1.001–1.079, *p* = 0.046), CA125 (Odds ratio 1.052, 95% CI 1.027–1.077, *p* < 0.001), CA153 (Odds ratio 1.073, 95% CI 1.042–1.105, *p* < 0.001), tumor size (Odds ratio 1.145, 95% CI 1.009–1.299, *p* = 0.035), vascular-invasion (Odds ratio 7.736, 95% CI 3.903–15.332, *p* < 0.001), calcification (Odds ratio 1.650, 95% CI 1.082–2.514, *p* = 0.02) and tumor grade (Odds ratio 5.338, 95% CI 1.514–18.840, *p* = 0.009 for grade 2, Odds ratio 5.864, 95% CI 1.670-20.594, *p* = 0.006 for grade 3) were independent prognostic factors for positive ALNs.

**Table 2:** Uni- and multivariate logistic regression analysis

**Variable**	**Univariate analysis**			**Multi-variate analysis**		
	Odds ratio	95% CI	*p*-value	Odds ratio	95% CI	*P* value
**Age**	0.852	0.733–0.999	0.036	0.993	0.975–1.011	0.435
**CEA**	1.282	1.188–1.384	< 0.001	1.085	1.001–1.079	0.046
**CA125**	1.057	1.036–1.078	< 0.001	1.052	1.027–1.077	< 0.001
**CA153**	1.124	1.094–1.154	< 0.001	1.073	1.042–1.105	< 0.001
**Tumor size**	1.313	1.168–1.475	< 0.001	1.145	1.009–1.299	0.036
**Location**						
** UOQ**	Reference					
** LOQ**	0.605	0.327–1.118	0.109			
** LIQ**	0.706	0.398–1.254	0.236			
** UIQ**	0.659	0.395–1.099	0.110			
** Central**	0.398	0.128–1.241	0.112			
**Vascular-invasion**						
** No**	Reference			Reference		
** Yes**	10.848	6.652–17.690	< 0.001	7.736	3.903–15.332	< 0.001
**Calcification**						
** No**	Reference			Reference		
** Yes**	1.900	1.325–2.271	< 0.001	1.650	1.082–2.514	0.02
**Tumor grade**						
** Well differentiated, grade 1**	Reference					
** Moderately differentiated, grade 2**	4.362	1.775–10.718	0.001	5.338	1.514–18.840	0.009
** Poorly differentiated, grade 3**	5.657	2.318–13.804	< 0.001	5.864	1.670–20.594	0.006
**Histology subtype**						
** Invasive ductal carcinoma**	Reference					
** Invasive lobular carcinoma**	1.838	0.755–4.477	0.180			
** Mixed**	2.056	0.964–4.379	0.062			
** Other**	0.503	0.215–4.379	0.113			
**ER status**						
** Negative**	0.693	0.447–1.073	0.101			
** Positive**	Reference					
**PR status**						
** Negative**	0.680	0.469–0.986	0.045			
** Positive**	Reference					
** KI67**	1.006	0.998–1.014	0.117			
**Her-2 status**						
** Negative**	0.920	0.626–1.352	0.673			
** Positive**	Reference					
**Biological subtype**						
** Luminal A**	1.147	0.532–2.474	0.726			
** Luminal B**	1.932	1.046–3.567	0.035			
** Her-2 positive**	1.554	0.706–3.421	0.273			
** TNBC**	Reference					

Abbreviation: *Her-2* Human epidermal growth factor receptor-2, *TNBC* Triple negative breast cancer, *LIQ* Lower-inner quadrant, *LOQ* Lower-outer quadrant, *UIQ* Upper-inner quadrant, *UOQ* Upper-outer quadrant

### Construction of predictive model

**Fig. 1:**
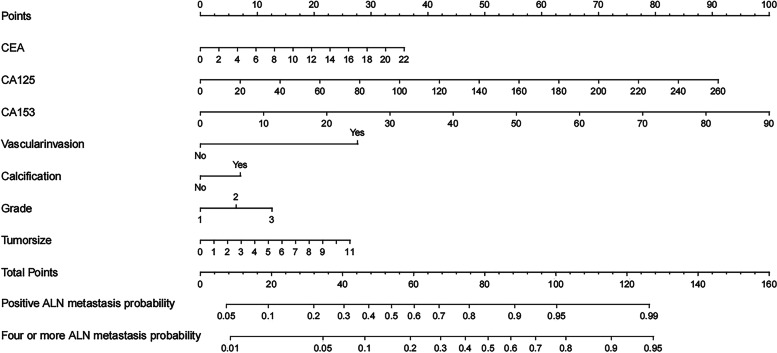
Nomogram for the prediction of positive ALN and four or more ALN metastasis. Abbreviations: ALN = axillary lymph node

All the significant factors were used to create the nomograms. The nomogram for positive ALN and four or more ALN metastasis was shown in Fig. 1. The value of each variable was given a score on the point scale axis. By adding up the scores related to each variable and projecting total scores to the bottom scales, it is easy to calculate the estimated probabilities for positive ALN and four or more ALN metastasis.

### Calibration and validation of nomogram

In order to identify the discriminating ability of nomograms, various methods were used in this study, including calibration curves, C-index values and DCA curves.

**Fig. 2:**
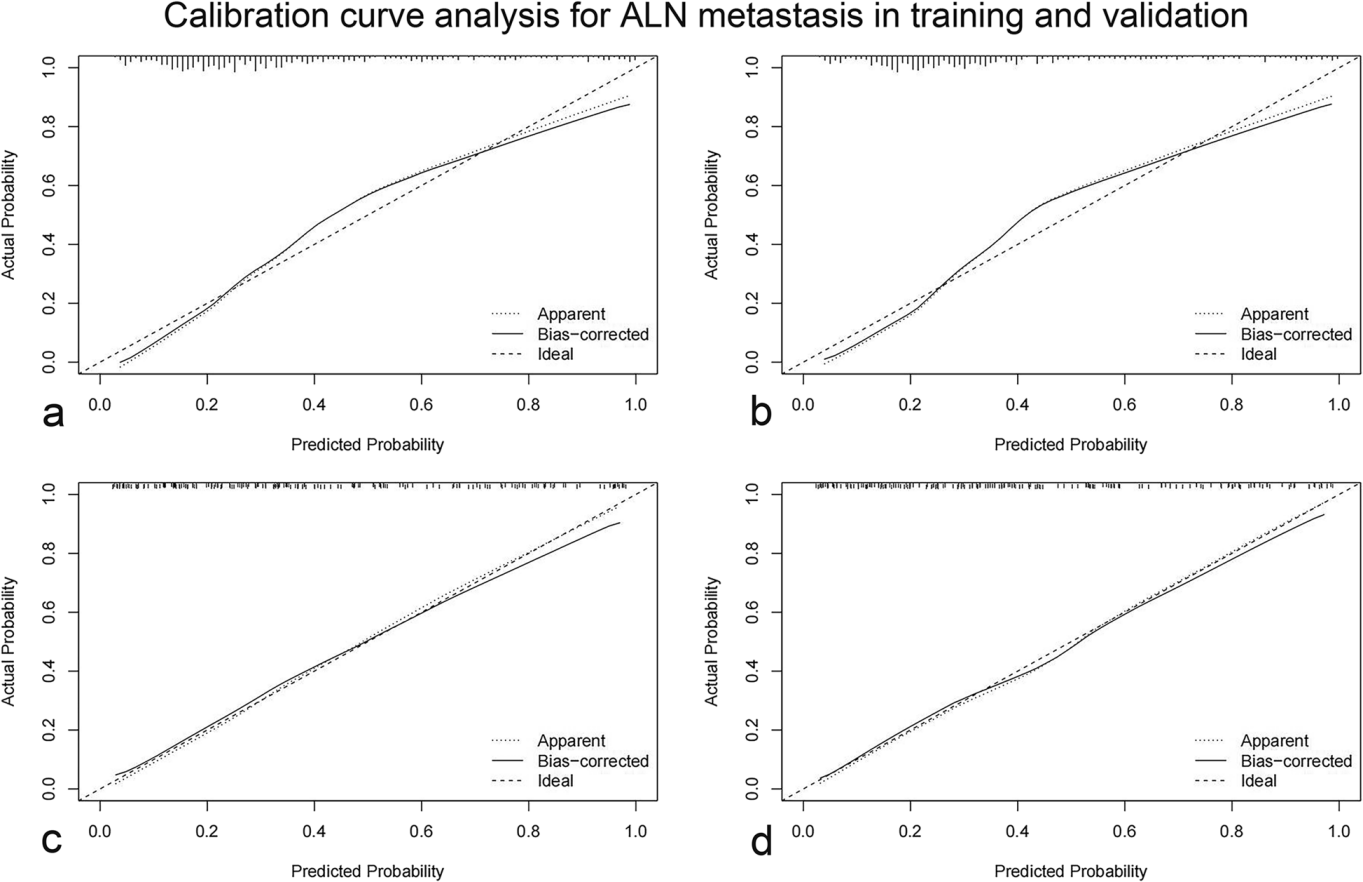
Calibration curve analysis for the prediction of positive ALN metastasis in training group (**a**), prediction of four or more ALN metastasis in training group (**b**), prediction of positive ALN metastasis in validation group (**c**), prediction of four or more ALN metastasis in validation group **d**. Abbreviations: ALN = axillary lymph node

**Fig. 3:**
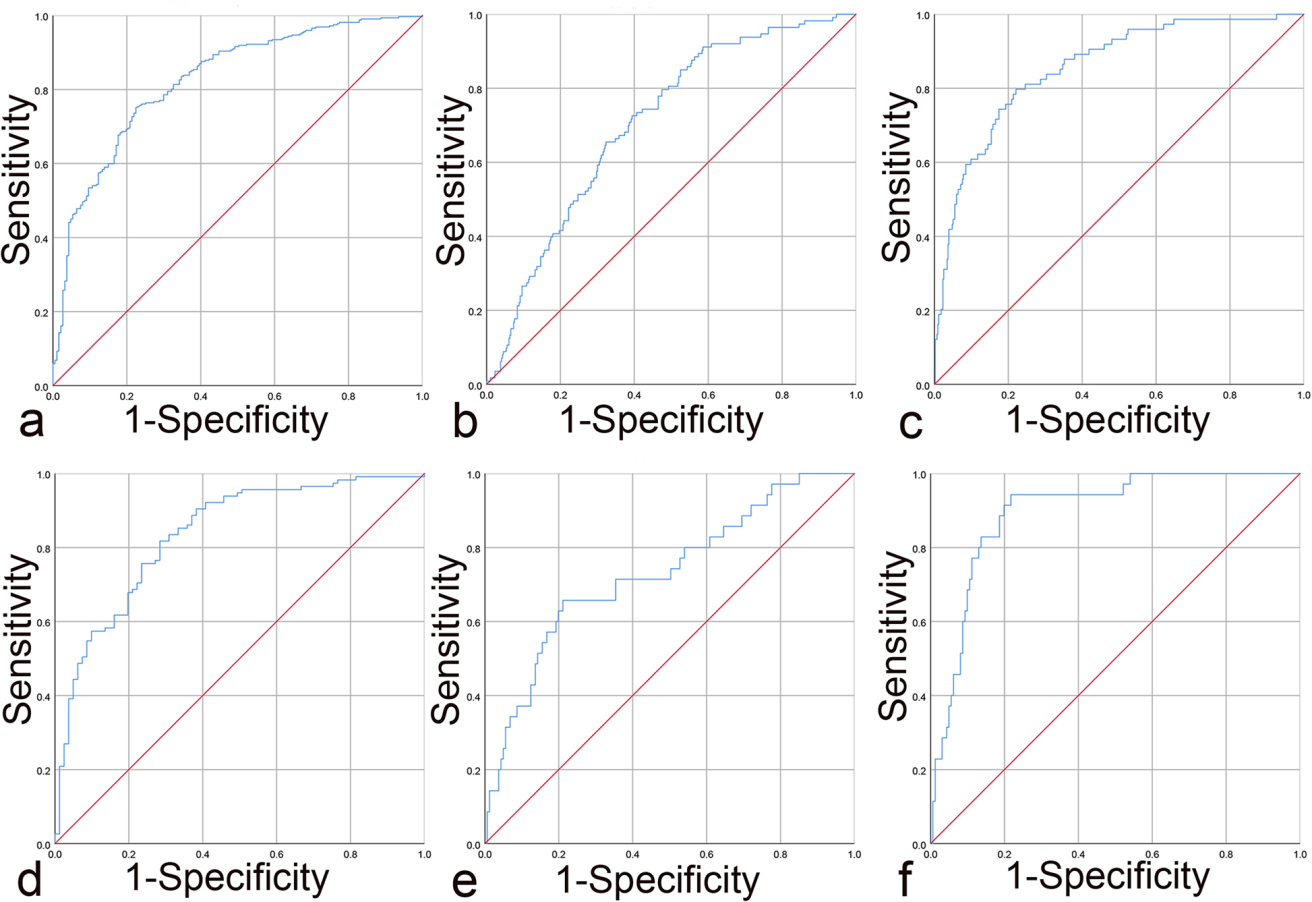
ROC curve analysis for prediction of no ALN metastasis in training group (**a**), prediction of positive ALN metastasis in training group (**b**), prediction of four or more ALN metastasis in training group (**c**), prediction of no ALN metastasis in validation group (**d**), prediction of positive ALN metastasis in validation group (**e**), prediction of four or more ALN metastasis in validation group **f**. Abbreviations: ROC = Receiver operating characteristic, ALN = axillary lymph node

**Fig. 4:**
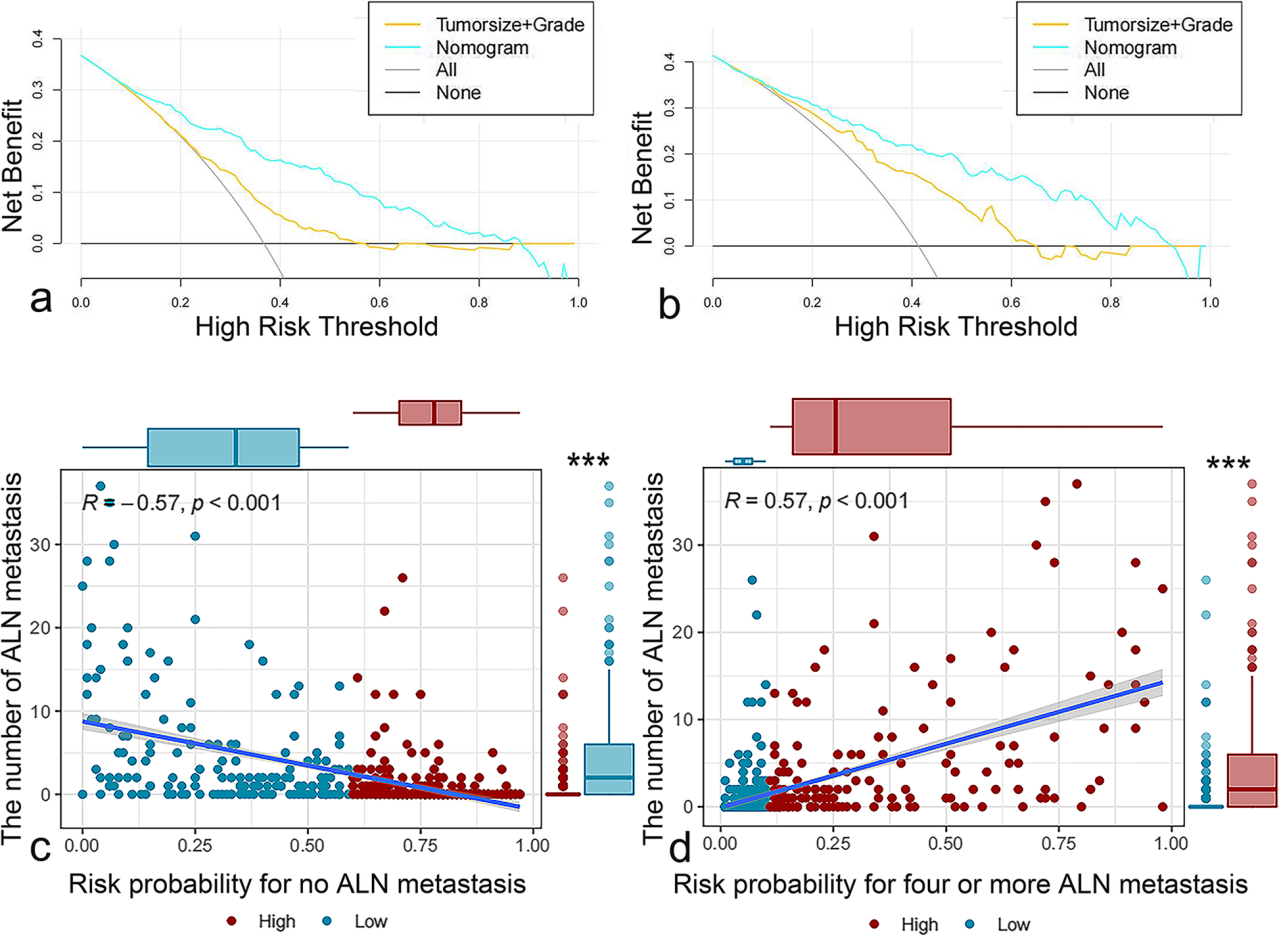
DCA analysis in training (**a**) and validation (**b**) group. Risk probability for no ALN metastasis (**c**) and for four or more ALN metastasis **d**. Risk grouping thresholds are 60% (c) and 10% (**d**). Abbreviations: ALN = axillary lymph node. DAC = decision curve analysis

In our study, calibration curves in the training group for positive ALN and four or more ALN metastasis were shown in Fig. 2a and b. There were no significant deviations between the calibration curves and the reference line, which revealed optimal agreement between model prediction and actual observations for ALN metastasis. In the validation group, the calibration curves for positive ALN and four or more ALN metastasis were shown in Fig. 2c and d. Similarly, good concordance was observed between the model predicted and the actual observations for ALN metastasis. The C-indexes of the nomogram for prediction of no ALN metastasis were 0.826 (95% CI 0.789–0.863, *p* < 0.001) in training group and 0.836 (95% CI 0.779–0.893, *p* < 0.001) in validation group, shown in Fig. 3a and d. The C-indexes of the nomogram for prediction of positive ALN were 0.706 (95% CI 0.656–0.756, *p* < 0.001) in the training group and 0.731 (95% CI 0.635–0.827, *p* < 0.001) in the validation group, showed in Fig. 3b and e. The C-indexes of the nomogram for prediction of four and more ALN metastasis were 0.855 (95% CI 0.809-0.900, *p* < 0.001) in the training group and 0.897 (95% CI 0.846–0.947, *p* < 0.001) in the validation group, showed in Fig. 3c and f. Moreover, in terms of the clinical utility, DCA analysis showed that our nomogram owned superior net benefits and net reduction in interventions. The nomogram was superior to the traditional staging system including tumor size and grade across the range of reasonable threshold probabilities in both the training group and the validation group, shown in Fig. 4a and b.

## Discussion

In this study, we constructed a novel, easy to use and effective nomogram for the prediction of positive ALN and four or more ALN metastasis. After univariant and multivariant logistic regression analysis, CEA (*p* = 0.046), CA125 (*p* < 0.001), CA153 (*p* < 0.001), tumor size (*p* = 0.035), vascular-invasion (*p* < 0.001), calcification (*p* = 0.02) and tumor grade (*p* = 0.009 for grade 2, *p* = 0.006 for grade 3) were incorporated as independent predictors of ALN metastasis in patients with breast cancer. In addition, according to the calibration curve and ROC curve analysis in both training group and validation group, the predictive accuracy and concordance of our nomogram were validated. What’s more, DCA analysis also displayed that our nomogram had better a clinical utility and more benefits than the traditional staging system.

ALN status is one of the most important prognostic factors for patients with breast cancer. For breast cancer patients with positive ALNs, it indicates the aggressiveness of the tumor, which may be related to their own biological characteristics. With the in-depth research on tumor molecular mechanisms and tumor microenvironment, the influence of tumor biological characteristics on the prognosis of patients was attached of great importance. Some studies had confirmed the vascular invasion’s ability to discriminate patients’ prognosis [40–42]. When concerning breast cancer, serum tumor markers including CEA, CA125, and CA15-3 and calcification had been revealed associated with patients’ clinical outcomes [31, 32, 35–37]. Previous researches [5–9] had tried to construct different nomograms to predict ALN status using patients’ traditional clinicopathological characteristics including tumor size, nuclear grade, and so forth, while most of them did not mention the role of tumor biological characteristics. To our knowledge, this is the first study to construct a nomogram incorporated patients’ clinicopathological and tumor biological characteristic factors. Our predictive model, based on previous studies and combined with tumor biomarkers, can better reflect the role of tumor biological characteristics in the progression of breast cancer. In addition, the results of the calibration curve and ROC curve analysis in both training group and validation group demonstrated the predictive ability and discrimination power of our model. Furthermore, combined the clinical utility with benefit shown in DCA analysis, our nomogram can be utilized effectively to counsel individual patients, thereby helping to personalize the surgical treatment of breast cancer patients.

In patients with positive SLN, present clinically useful predictive models, including the well-known MSKCC nomogram [10] were explicitly designed to predict the non SLN metastasis with patients’ clinicopathological factors. In patients with negative SLN, ALND can safely be avoided. However, M. Klar et Al’s study [43, 44] had shown that side-effects of SLN biopsy without consecutive ALND are not negligible. Furthermore, considering the unreliable results of SLNB caused by the false negative rate [17–21] and lower incidence of axillary nodal metastasis at the time of diagnosis [22] due to preoperative examination technique improvement, the benefits of SLNB were reducing. Our novel predictive model incorporated the patient’s clinicopathological and tumor biological characteristics and has better functionality and accuracy. First, our nomogram was proved to be capable of defining 8% of patients as having low-risk (lower than 10% chance) of ALN metastasis (showed in Fig. 4c). The predictive accuracy was high in both training group and validation group (ROC analysis, 0.826, 95% CI 0.789–0.863, *p* < 0.001 in the training group and 0.836, 95% CI 0.779–0.893, *p* < 0.001 in the validation group). Second, our nomogram had the ability to define 68.5% of patients as having a low-risk (lower than 10% chance) of four or more ALN metastasis (showed in Fig. 4d). Similarly, the ROC analysis revealed good prediction sensitivity and specificity (ROC analysis, 0.855, 95% CI 0.809-0.900, *p* < 0.001 in the training group and 0.897, 95% CI 0.846–0.947, *p* < 0.001 in the validation group). In addition, with the capability to predict breast cancer patients with less than four positive ALN metastasis, our model can improve the utilization efficiency of SLNB and reduce the incidence of complications. At the same time, according to the existing research [11–14], it can also provide patients with better clinical treatment decisions based on the results of SLNB.

Some limitations in the present study needs to be considered. Firstly, the number of patients in the validation group enrolled in this study was relatively small. In order to better evaluate and validate the nomogram, more patients need to be enrolled in the study from multiple centers to improve the credibility of the study. Secondly, not all patients in this study underwent ALND. Therefore, it is possible that non-SLN metastasis may have remained. Thirdly, we would incorporate more indexes including the results of images (like mammography or MRI) of axillary lymph node status to improve the establishment of the prediction model in further research.

## Conclusion

we successfully construct and independently validate the nomogram to predict patients’ axillary node status by using patients’ clinicopathological and tumor characteristic factors. Furthermore, we sought the possibility to find selected patients to perform SLNB, it would decrease the time of surgery and reduce the postoperative complications, which can give patients better clinical treatment.

## Data Availability

The dataset of the current study was available from the corresponding author on reasonable request.

## Abbreviations

Her-2: Human epidermal growth factor receptor-2
TNBC: Triple negative breast cancer
ALND: Axillary lymph node dissection
SLNB: Sentinel lymph node biopsy
ER: Estrogen receptor
PR: Progesterone receptor
DCA: Decision curve analysis
ROC: Receiver operating characteristic curve
BCS: Breast conserving surgery
FNR: False negative rate
